# From Concept to Cure: The Life and Legacy of Scipione Riva-Rocci

**DOI:** 10.7759/cureus.70436

**Published:** 2024-09-29

**Authors:** Khushman K. Bhullar, Narayanpreet Singh

**Affiliations:** 1 Internal Medicine, Sri Guru Ram Das Institute of Medical Sciences and Research, Amritsar, IND; 2 Medicine, Sri Guru Ram Das Institute of Medical Sciences and Research, Amritsar, IND

**Keywords:** blood pressure, cardiovascular diseases, hypertension, riva rocci, sphygmomanometer

## Abstract

The evolution of cardiovascular diagnostics has been profoundly influenced by the invention of the sphygmomanometer, a groundbreaking tool that transformed the measurement of blood pressure from an imprecise and invasive process into a routine and reliable clinical practice. Scipione Riva-Rocci, an Italian physician, developed the mercury sphygmomanometer in 1896, revolutionizing the way blood pressure was measured and managed. This simple yet innovative device, comprising an inflatable cuff, a mercury column manometer, and a stethoscope, provided the first accurate, noninvasive method for measuring blood pressure. The introduction of the Riva-Rocci sphygmomanometer not only enhanced the ability to diagnose and treat hypertension but also laid the foundation for modern cardiovascular medicine. The widespread adoption of this device has had a lasting impact on patient care, enabling early detection and management of cardiovascular diseases, thus significantly reducing morbidity and mortality. This review examines the design, development, and enduring legacy of Riva-Rocci’s sphygmomanometer, highlighting its pivotal role in the history of medical innovation and its continued relevance in contemporary clinical practice.

## Introduction and background

**Figure 1:**
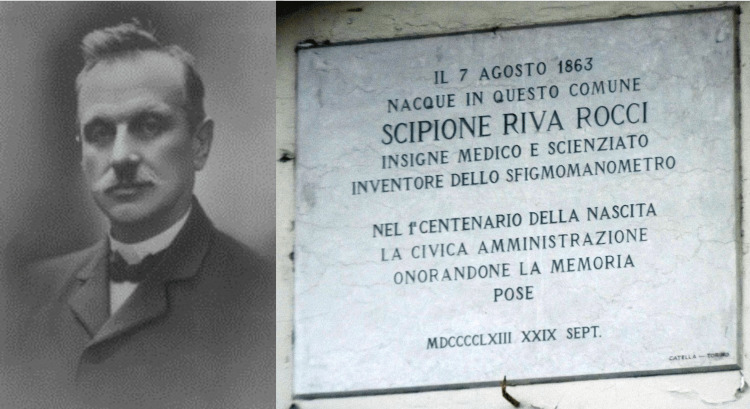
Scipione Riva-Rocci (1863-1937) Images in the public domain under Creative Commons License [6,7]

Scipione Riva-Rocci (1863-1937), an Italian physician (Figure 1) and inventor, is a pivotal figure in the annals of medical history, notably for his revolutionary contribution to cardiovascular diagnostics - the mercury sphygmomanometer. Born in Almese, Italy, Riva-Rocci pursued his medical education at the University of Turin, where he graduated in 1888 and spent his first postgraduate years in the Institute of Clinica Medica. He then became an assistant to Professor Carlo Forlanini (1847-1918), who was known for his treatment of pulmonary tuberculosis by a deliberately induced pneumothorax [1,2].

His analytical and innovative thinking was highly evident, and he played a significant role in the advancement of the artificial pneumothorax. One of the most effective treatment approaches that was employed for many years in the fight against tuberculosis grew out of these investigations. He developed a strong proficiency with regulated pressures thanks to these investigations [2,3]. After that, he developed an interest in the challenge of noninvasive blood pressure measurement, which had long eluded researchers despite their best efforts. In 1896, he presented "Un nuovo sfigmomanometro" (a new sphygmomanometer) at the Accademia Medica Reale di Torino, based on his research on the topic. His early career in internal medicine and pediatrics laid the foundation for his later groundbreaking invention, which would transform the blood pressure measurement landscape.

Prior to Riva-Rocci's innovation, methods for measuring blood pressure had been fraught with limitations, ranging from invasive procedures to unreliable techniques. Riva-Rocci produced four publications on a novel method of measuring blood pressure in 1896 and 1897. All measurements had been made on the A. radialis up until that point, initially using blood and then indirectly. Because physicians had been attempting to gauge the strength of the circulation there for about 20 centuries, the liveliness of the pulse was seen to be just as significant a diagnostic indicator as its frequency. Riva-Rocci extensively described the existing methods for measuring pulse pressure, all so cumbersome and unreliable that modifications were constantly being proposed [1,2].

Before publishing his method, Riva-Rocci conducted several experiments. He calibrated his procedure, for instance, on deceased people by opening the A. radialis and attaching the A. axillaris to a water reservoir via a riser [3,4]. Additionally, subsequent measurements on living participants nearly always produced the same results (around 125 mm of mercury), with the exception of feelings or arm muscular contractions. Since he had seen extremely high pressures in individuals with severe kidney illnesses or retinal hemorrhages, Riva-Rocci instantly made references to clinical uses [5].

Recognizing the critical need for a more accurate and noninvasive approach, Riva-Rocci introduced the mercury sphygmomanometer in 1896. This device, consisting of an inflatable cuff, mercury column manometer, and stethoscope, enabled clinicians to obtain precise systolic and diastolic blood pressure readings through a standardized, reproducible method - the Riva-Rocci method. The significance of Riva-Rocci's invention transcended mere technological advancement; it revolutionized clinical practice by facilitating early detection and management of hypertension, thereby mitigating the risks of stroke, heart disease, and other cardiovascular disorders. Despite initial challenges in gaining widespread recognition during his lifetime, Riva-Rocci's legacy has endured through ongoing adaptations and advancements in blood pressure monitoring technology.

## Review

Concept and design of the first mercury sphygmomanometer

The concept and design of the first mercury sphygmomanometer, developed by Scipione Riva-Rocci in 1896, marked a significant milestone in the history of medical instrumentation. Prior to Riva-Rocci's innovation, methods for measuring blood pressure had been limited and often inaccurate, relying on palpation or more invasive techniques.

Conceptualization

**Figure 2:**
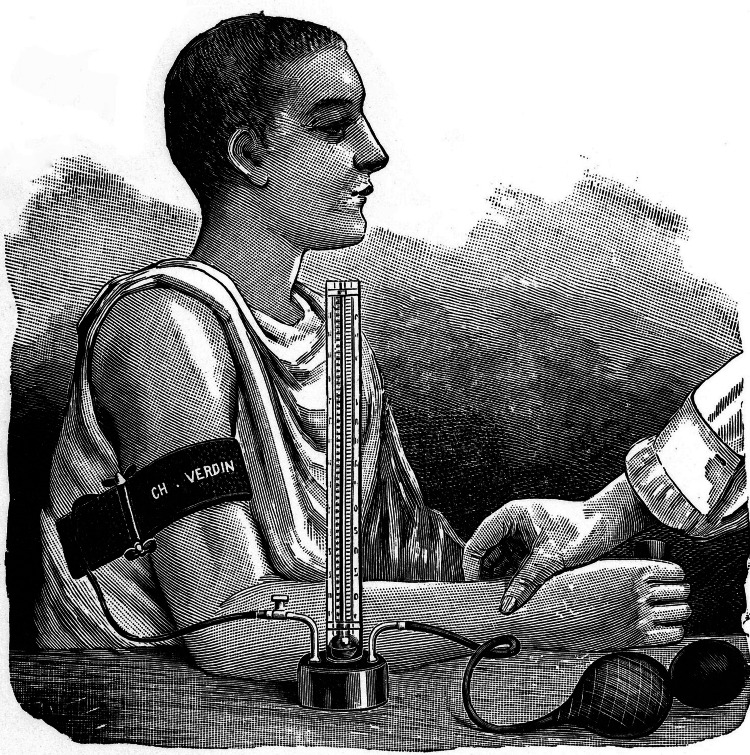
Riva-Rocci's syphgmomanometer in use Image in the public domain under Creative Commons License [8]

Riva-Rocci envisioned a device that could accurately measure blood pressure in a noninvasive manner, providing clinicians with reliable data for diagnosing and managing cardiovascular conditions. His goal was to develop a method that was both precise and accessible, enhancing the standard of care in medical practice. His novel technique involved delivering the pressure necessary to cause the radial pulse to vanish on the upper arm rather than the wrist itself, and measuring it at the same time. He employed a "cuff" for this purpose, which was made of a bicycle inner tube that is 4-5 cm wide and is secured with an adjustable clamp. Because the cuff was wrapped with a sturdy fabric on the exterior, it could only expand inward [1]. Two balloons were used to raise the air pressure until the pulse could no longer be felt. The manometer was situated between the balloons and the cuff (Figure 2), ideally with a mercury column.

Design Features

The mercury sphygmomanometer designed by Riva-Rocci consisted of several key components:

Inflatable cuff: A cuff made of fabric or rubber that could be wrapped around the patient's arm and inflated to constrict the brachial artery [1-2].

Mercury manometer: A vertical glass tube filled with mercury, connected to the cuff via tubing. The height of the mercury column varied with changes in pressure within the cuff, providing a numerical measurement of systolic blood pressure. Riva-Rocci measured the peak (systolic) blood pressure by observing the cuff pressure at which the radial pulse was no longer palpable. If one closely examines Riva-Rocci's writings, one finds that he was also interested in what is now known as "mean blood pressure." He defined "lateral pressure" and tried to estimate it by using the sphygmomanometer as a parathlibometer, i.e. as an oscillometer, measuring "lateral pressure" while maximal oscillations of the pulse could be seen or recorded [4,5].

**Figure 3:**
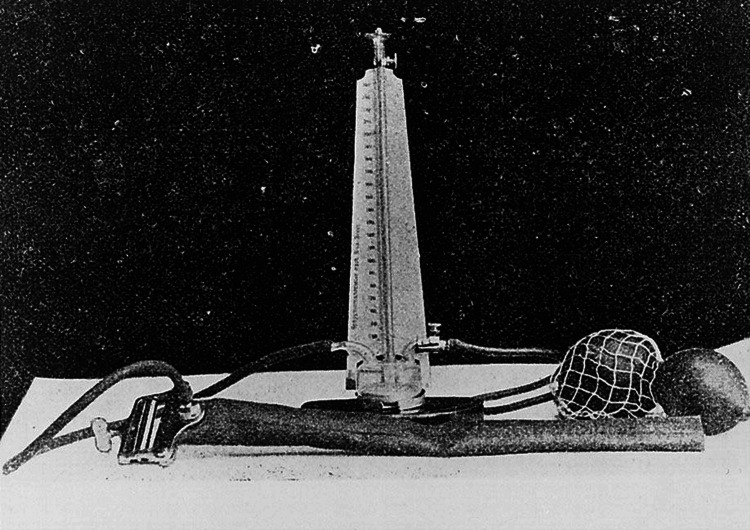
Riva-Rocci's sphygmomanometer used by Korotkoff in his measurements The length of the cuff was approximately 1 2 arshin (35.56 cm) and the width was at least 21 2 to 3 inches (6.35-7.62 cm) Image in the public domain under Creative Commons License [9]

Stethoscope: To determine the diastolic blood pressure, Riva-Rocci tried to feel when the pulse was at its maximum strength again, but this method was not very reliable. In 1905, the then 31-year-old surgeon Nikolai Korotkoff (1874-1920) described to a critical audience of military physicians in St. Petersburg how he solved this problem by auscultating the artery under the cuff as per which, if the pressure in the cuff is increased until the pulse wave tones have disappeared and then reduced, the appearance of the first pulse wave tones corresponds to the systolic pressure, and their disappearance to the diastolic pressure; the latter means that the pulse wave can then pass unhindered (Figure 3).

Operating principle

The operation of the modern-day sphygmomanometer is based on the Riva-Rocci method, supplemented by findings of Nikolai Korotkoff, involving several steps:

Cuff Inflation

The cuff was inflated to a pressure higher than the systolic pressure, temporarily occluding the brachial artery [2,3].

Gradual Deflation

Pressure within the cuff was slowly released while simultaneously listening with a stethoscope placed over the brachial artery [4-5].

Detection of Sounds

As the cuff pressure decreased, blood flow resumed through the artery, producing distinct sounds known as Korotkoff sounds. The first sound heard (Korotkoff Phase I) indicated the systolic pressure, while the disappearance of sound (Korotkoff Phase V) indicated the diastolic pressure.

How the sphygmomanometer revolutionized cardiovascular health

The introduction of the Riva-Rocci sphygmomanometer led to the standardization of blood pressure measurement across medical practices worldwide. The method was simple enough to be adopted by physicians in various settings, from hospitals to general practice. This standardization ensured that blood pressure readings were consistent and comparable, which was crucial for the diagnosis and treatment of hypertension. With the advent of accurate blood pressure measurement, the medical community gained a powerful tool for managing cardiovascular diseases. Hypertension, often referred to as the "silent killer" due to its asymptomatic nature, could now be effectively monitored and controlled. This led to a significant reduction in the incidence of stroke, heart attack, and other complications associated with high blood pressure. The ability to measure and monitor blood pressure also paved the way for the development of antihypertensive medications, further improving patient outcomes.

The widespread use of the sphygmomanometer had a profound impact on public health. Regular blood pressure screenings became a standard part of medical care, leading to increased awareness of hypertension among both healthcare providers and the general public. This awareness contributed to the development of public health initiatives aimed at reducing the prevalence of hypertension through lifestyle modifications, such as diet and exercise, and the use of medications. Riva-Rocci’s invention laid the groundwork for future advancements in cardiovascular health. The principles behind the sphygmomanometer have been refined and adapted over the years, leading to the development of more advanced blood pressure measurement devices, including electronic sphygmomanometers and automated blood pressure monitors. These innovations have further improved the accuracy and ease of blood pressure measurement, making it even more accessible to both healthcare professionals and patients.

Harvey Cushing and Global Impact

Scipione Riva-Rocci’s invention of the mercury sphygmomanometer in 1896 was a milestone in medical history, providing the first reliable method for noninvasive blood pressure measurement. However, the widespread adoption of this crucial medical tool was not instantaneous. It took the efforts of several influential physicians to bring Riva-Rocci's innovation to the global stage. Among these was American neurosurgeon Harvey Cushing (1869-1939), a pioneer whose work was key in popularizing the sphygmomanometer in the United States and beyond. Cushing's advocacy for the device not only revolutionized the measurement of blood pressure but also significantly impacted clinical practices, particularly in surgery and internal medicine. In 1901, Cushing visited Riva-Rocci in Pavia, where he did sketches and received a model of his apparatus [2]. After returning to the United States, he created an enhanced version of the same device and successfully used it at Johns Hopkins Hospital, mostly for intracranial surgery. Cushing was a major force behind the popularization of Riva-Rocci's mercury sphygmomanometer, with assistance from Theodore Janeway in New York City and George Crile in Cleveland. 

While Riva-Rocci’s original design was effective, Cushing made several modifications to improve its usability in a clinical setting. He collaborated with instrument makers to create a more portable and durable version of the sphygmomanometer, which could be used in various medical settings, from operating rooms to general practice. One of his key contributions was the introduction of a wider cuff, which provided more accurate readings in larger patients [10,11].

The adoption of the sphygmomanometer in the United States had a profound impact on both surgical and medical practices. In surgery, the ability to monitor blood pressure accurately allowed for safer anesthetic management and better outcomes. In internal medicine, it enabled the early detection and management of hypertension, which had been previously difficult to diagnose. Cushing’s efforts helped to establish blood pressure measurement as a routine part of patient care, revolutionizing the management of cardiovascular health [12].

It is imperative to note that Riva-Rocci did not profit financially from his innovation and consistently declined to have it patented. 

## Conclusions

Scipione Riva-Rocci's invention of the mercury sphygmomanometer marked a turning point in the history of medicine. By providing a reliable and noninvasive method for measuring blood pressure, Riva-Rocci enabled the early detection and effective management of hypertension, reducing the burden of cardiovascular diseases worldwide. Although he did not achieve widespread recognition during his lifetime, Riva-Rocci's legacy endures through the continued use of blood pressure measurement tools that have evolved from his original design. His work exemplifies the profound impact that innovation can have on clinical practice and patient outcomes, highlighting the enduring link between scientific discovery and improved healthcare.
